# Heart University: a new online educational forum in paediatric and adult congenital cardiac care. The future of virtual learning in a post-pandemic world?

**DOI:** 10.1017/S1047951120000852

**Published:** 2020-04-13

**Authors:** Justin T Tretter, Jonathan Windram, Theresa Faulkner, Michelle Hudgens, Skaiste Sendzikaite, Nico A Blom, Katarina Hanseus, Rohit S Loomba, Colin J McMahon, Bistra Zheleva, Raman Krishna Kumar, Jeffrey P Jacobs, Erwin N Oechslin, Gary D Webb, Andrew N Redington

**Affiliations:** 1 The Heart Institute, Cincinnati Children’s Hospital Medical Center, Cincinnati, OH, USA; 2 Department of Pediatrics, University of Cincinnati College of Medicine, Cincinnati, OH, USA; 3 Mazankowski Alberta Heart Institute, University of Alberta Hospital, Edmonton, Alberta, Canada; 4 Institute of Clinical Medicine, Vilnius University, Vilnius, Lithuania; 5 Division of Pediatric Cardiology, Leiden University Medical Center, Leiden, the Netherlands; 6 Division of Pediatric Cardiology, Academic Medical Center, Amsterdam, the Netherlands; 7 Department of Paediatric Cardiology, Skane University Hospital, Lund, Sweden; 8 Division of Cardiology, Advocate Children’s Hospital, Oak Lawn, IL, USA; 9 Department of Paediatric Cardiology, Our Lady’s Children’s Hospital, Crumlin, Dublin, Ireland; 10 Children’s HeartLink, Minneapolis, MN, USA; 11 Department of Pediatric Cardiology, Amrita Institute of Medical Sciences and Research Centre, Kochi, Kerala, India; 12 Cardiology in the Young, Cambridge, UK; 13 Toronto Congenital Cardiac Centre for Adults at Peter Munk Cardiac Centre, University Health Network, University of Toronto, Toronto, Ontario, Canada

**Keywords:** Adult CHD, CHD, medical education, online learning

## Abstract

Online learning has become an increasingly expected and popular component for education of the modern-day adult learner, including the medical provider. In light of the recent coronavirus pandemic, there has never been more urgency to establish opportunities for supplemental online learning. Heart University aims to be “the go-to online resource” for e-learning in CHD and paediatric-acquired heart disease. It is a carefully curated open access library of paedagogical material for all providers of care to children and adults with CHD or children with acquired heart disease, whether a trainee or a practising provider. In this manuscript, we review the aims, development, current offerings and standing, and future goals of Heart University.

The personal computer entered the market in 1977 and became widely popular and accessible for the non-technical user throughout the 1980s, but it was the establishment of the World Wide Web (WWW) in 1989 that revolutionised “connectivity” and changed our daily lives.^1^ However, it was the introduction of smart phones at the turn of the century that further changed the world. By 2013, over 1 billion consumers worldwide owned some form of a smart phone.^2^ With the subsequent advent and infiltration of social media into the backbone of modern-day culture, the fundamental right of freedom of speech has both been challenged and solidified and, for better or worse, has facilitated exponentially “freedom of reach”.

Almost every aspect of our lives has been affected by these incredible advances, not least the way we educate and learn. Millennial medical learners (those born 1981–2000) have grown up with personal computers and smartphones and have quite different expectations for the way they train and learn.^3^ For example, in a recent online survey of adult CHD trainees, respondents stated they were more likely to search information online (58%) than consult a faculty member (29%) or textbook (3%). Over two-thirds of respondents stated they used their smartphones at least once daily to search for information during regular clinical work.^4^ In light of the current coronavirus pandemic, with mass cancellations of international, national, and regional conferences as well as institutional grand rounds and weekly trainee lectures, the world of academic medicine is rethinking how to distribute new scientific ideas and maintain the necessary ongoing education of trainees and practising physicians.^5^

To meet these changing educational needs and desires, e-learning programmes are now becoming increasingly more commonplace in higher education, including in continuing medical education. However, there remain significant barriers to implementing online learning programmes. Attitudinal barriers cannot be underestimated when considering both those involved in teaching (a nostalgia for the classroom), as well as sometimes in the learner (some modern learners still prefer the printed page). However, more and more lectures are now recorded for subsequent online posting. While these cannot replace textbooks, such recorded lectures are supplementary and are becoming the norm for many university-based graduate and postgraduate degree courses. That said, we do need to look at education through a worldwide lens, and while access is growing in almost all countries, consistent quality education remains a barrier for those particularly in most low- and middle-income regions. This is especially true regarding specialty and sub-specialty education.^6,7^ These barriers often have identifiable solutions.^8^ For instance, even in areas with limited access to computers and poor physical infrastructure, a break-even cost analysis by Maloney and colleagues^9^ found that the web-based approach to learning was superior to the traditional face-to-face education. Consequently, when resources are limited, investment in e-learning may, paradoxically, be a cheaper solution than a “bricks and mortar” approach.

Not all learning is the same, however, and the unique imperatives of medical education (practitioner–patient communication, physical examination, etc.) can never be replaced completely by online approaches. Furthermore, while some studies have shown benefit in e-learning for medical education when compared to traditional learning, a recent Cochrane review of the limited randomised controlled trials comparing e-learning to traditional learning for medical education concluded that e-learning may make little or no difference in patient outcomes or health professionals’ behaviours, skills, or knowledge.^10^ Nonetheless, “non-inferiority” of e-learning is probably adequate enough to sustain and expand this now-expected approach of today’s learners. There is likely no going back, and so looking forward we should be customising and enhancing the e-learning experience, a philosophy that was the bedrock of the formation of Heart University (Fig 1).

**Figure 1.**
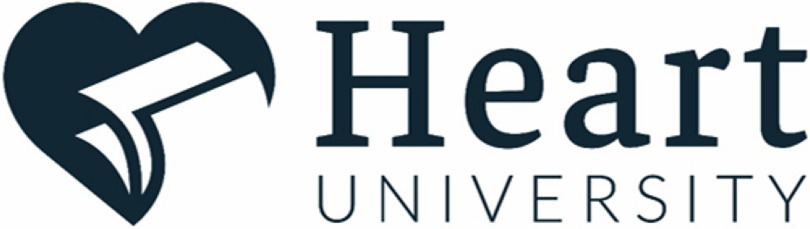
Heart University logo.

## Aims of Heart University

Heart University aims to be “the go-to online resource” for e-learning in CHD and paediatric-acquired heart disease. It is a carefully curated open access library of educational material for all providers of care to children and adults with CHD or children with acquired heart disease, whether a trainee or a practising provider. The site provides free content to a global audience in two broad domains:

1. A comprehensive curriculum of training modules and associated testing for trainees.
2. A curated library of conference and grand rounds recordings for continuing medical education.

## Development of Heart University

In 2012, under the leadership of Gary Webb and with the support of Cincinnati Children’s Hospital Medical Center and the International Society for Adult Congenital Heart Disease (https://isachd.connectedcommunity.org/home), a web-based learning platform for providers of adult CHD was created, the *ACHD Learning Center*. The original aim was to provide open access to high-quality educational resources for cardiology trainees, cardiologists, and healthcare professionals interested in learning more about adult patients with CHDs. These aims were supported by both an expressed need from providers for further instruction to care for such patients effectively,^11^ as well as the understanding that poorer outcomes in adults with CHD are associated with care provided by general cardiologists who are not educated in caring for these patients.^12^ The ACHD Learning Center rapidly outperformed its most optimistic expectations. From its inception in 2012 up to the creation of Heart University in the spring of 2017, the ACHD Learning Center had accrued over 2000 users from 36 countries, including 42 academic programmes requiring use of the training modules by their trainees, with over 10,000 self-assessment tests taken.

In the spring of 2017, given the success of the ACHD Learning Center, a team was assembled to develop a corresponding educational website geared towards providers taking care of children with acquired or CHD, the *Pediatric Cardiac Learning Center*. At the same time, it was recognised that the ACHD Learning Center could be enhanced by a modular learning system, prescribed teaching courses, and self-assessment tests to better serve the modern learner. These two sites would live under the parent site, Heart University (www.heartuniversity.org).

### Learning management system

**Figure 2.**
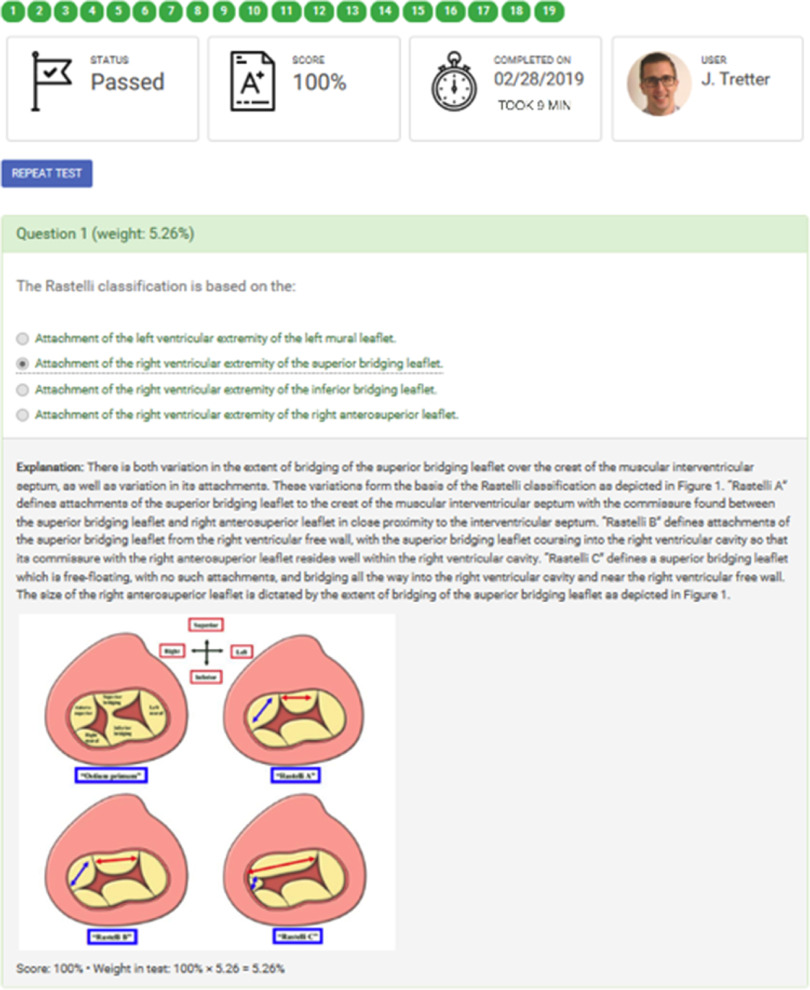
The eFront Learning Management System offers excellent testing capabilities, analytics, and user feedback.

The initial platform for the ACHD Learning Center did not provide all the necessary features for detailed analytics of site usage, nor did it provide an engaging interface for learning modules and associated testing. The initial goal of the Heart University team was to select the appropriate learning management system that had all the desired features to fulfil the stated aims. A learning management system is a software application for maintaining, delivering, and tracking educational resources.^13^ To fulfil the global, open access aims of Heart University, the team selected the eFront learning management system, created by DHx Software, LLC. This system comprehensively met the outlined needs, with the necessity of secure user login protocols, easy-to-navigate and visually appealing user interface, and detailed background analytics and learning assessment (Fig 2). The detailed design of the Heart University learning management system began in November 2017, with initial focus on developing the newly conceived Pediatric Cardiac Learning Center and transitioning the existing ACHD Learning Center over to the new learning management system.

### Site organisation and navigation

**Figure 3.**
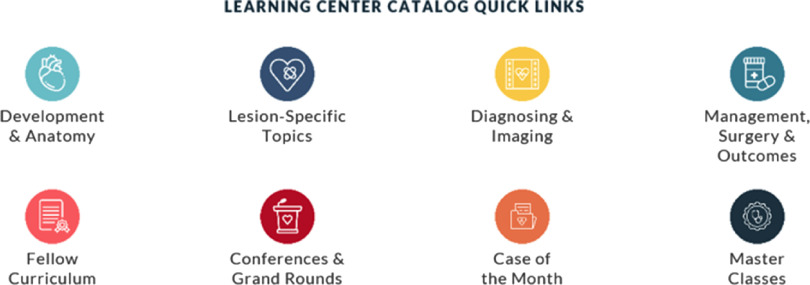
Content organisation of both the Pediatric Cardiac Learning Center and ACHD Learning Center.

The initial focus was concise organisation, clear and concise labelling, visual appeal, and easy navigation (Fig 3). Putting oneself in the shoes of the user is a key component to optimising the user’s experience. This process was a concerted effort between the site editors, website and graphic designers, and the eFront learning management system development team.

For both the Pediatric Cardiac Learning Center and ACHD Learning Center, the general organisational scheme was structured under four main categories:

1. Development and Anatomy
2. Lesion-specific Topics
3. Diagnosing and Imaging
4. Medical management, Surgery, and Outcomes

The four main categories were further sub-categorised to optimise the organisation of content and navigability. This strategy allows users to find with ease the relevant content, through multiple avenues. For example, in addition to a general search, if a user was interested in finding recorded conference material covering echocardiographic imaging of patients with atrioventricular septal defects, they could find this material through two general pathways:

Option #1: Lesion-specific Topics → Atrioventricular Septal Defect/Atrioventricular Canal → Diagnosis and Imaging
Option #2: Diagnosis & Imaging → Echocardiography → Lesion-specific Lectures

**Figure 4.**
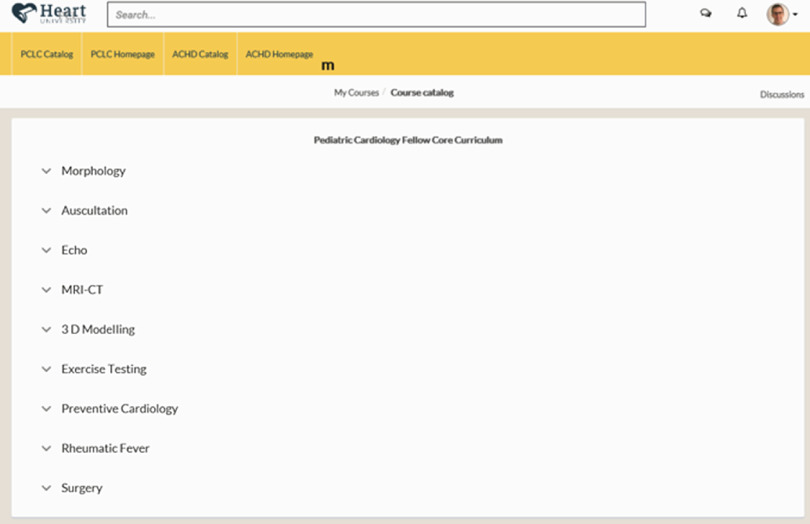
After clicking on the “Fellow Curriculum” icon from Figure 3 when within the Pediatric Cardiac Learning Center, the user is brought to the Pediatric Cardiology Fellow Core Curriculum.

If the user was primarily interested in working through the Pediatric Cardiology Fellow Core Curriculum, with learning modules and associated test questions, the user would click directly into the Fellow Curriculum (Figs 3 and 4), whereas a user primarily interested in continued medical education might select international or national conference or grand rounds material, by clicking on Conferences & Grand Rounds (Fig 4).

**Figure 5.**
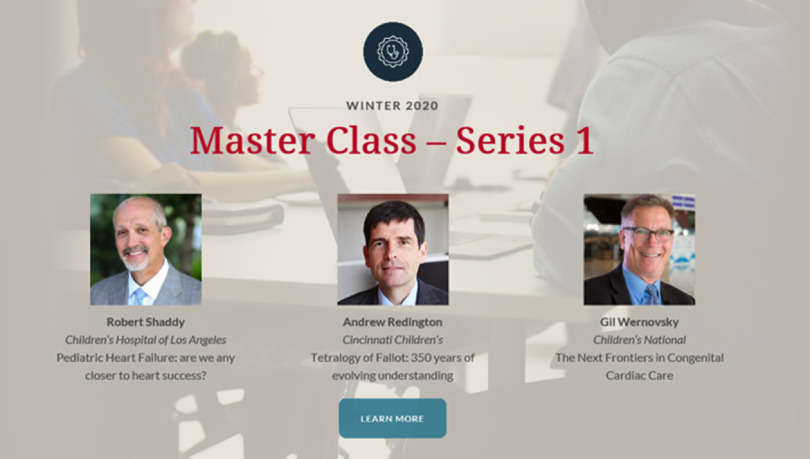
The Master Class Series.

**Figure 6.**
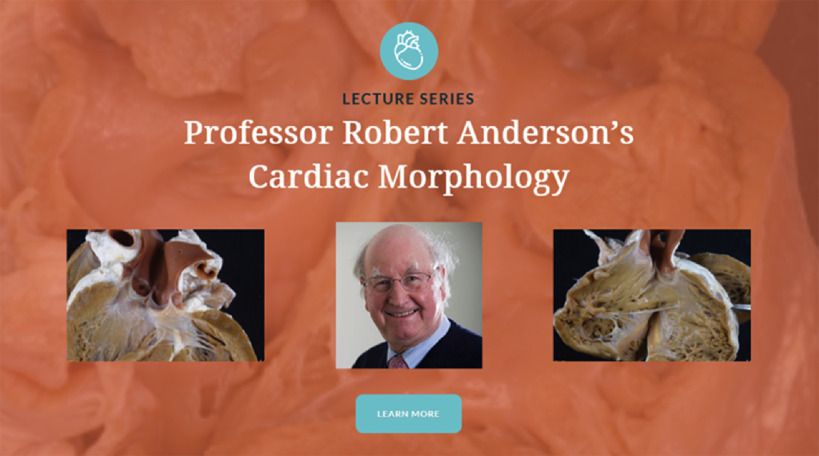
Professor Robert Anderson’s Cardiac Morphology Lecture Series.

**Figure 7.**
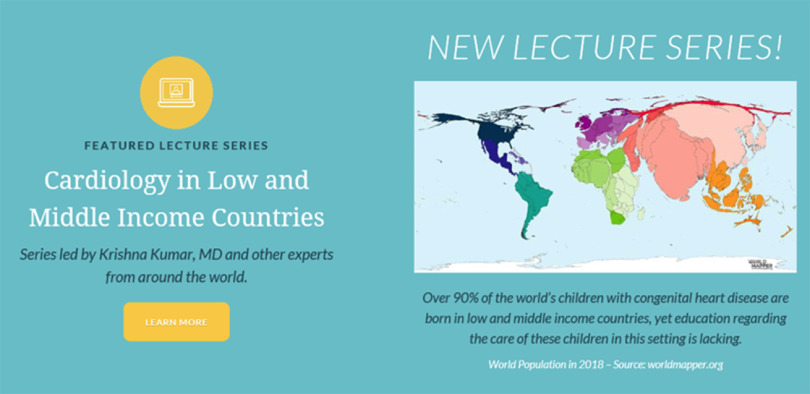
Cardiology in Low- and Middle-Income Countries Lecture Series.

For the non-directed user, we have produced a slider at the top of both the Pediatric Cardiac Learning Center and ACHD Learning Center which highlight featured material unique to the website. Examples of this slider include the following:

1. **Master Class**: a series of landmark lectures released at set intervals given by experts in the field of congenital and acquired paediatric heart disease, covering both the pertinent history and development of current understanding which has led to improved management and outcomes (Fig 5).
2. **Professor Robert Anderson’s Cardiac Morphology Lecture Series**: a collection of recorded lectures given by renowned cardiac morphologist, Professor Robert Anderson, covering a comprehensive curriculum of normal and abnormal cardiac development and anatomy (Fig 6).
3. **Cardiology in Low- and Middle-Income Countries**: a developing lecture series led by Dr Krishna Kumar, with the endorsement from Children’s HeartLink, with invited guest speakers covering topics specific to the management of children and adults with CHD in low- and middle-income countries (Fig 7).
4. **Case of the Month**: submitted case reports featuring interesting cases, providing an open forum for discussion.

**Figure 8.**
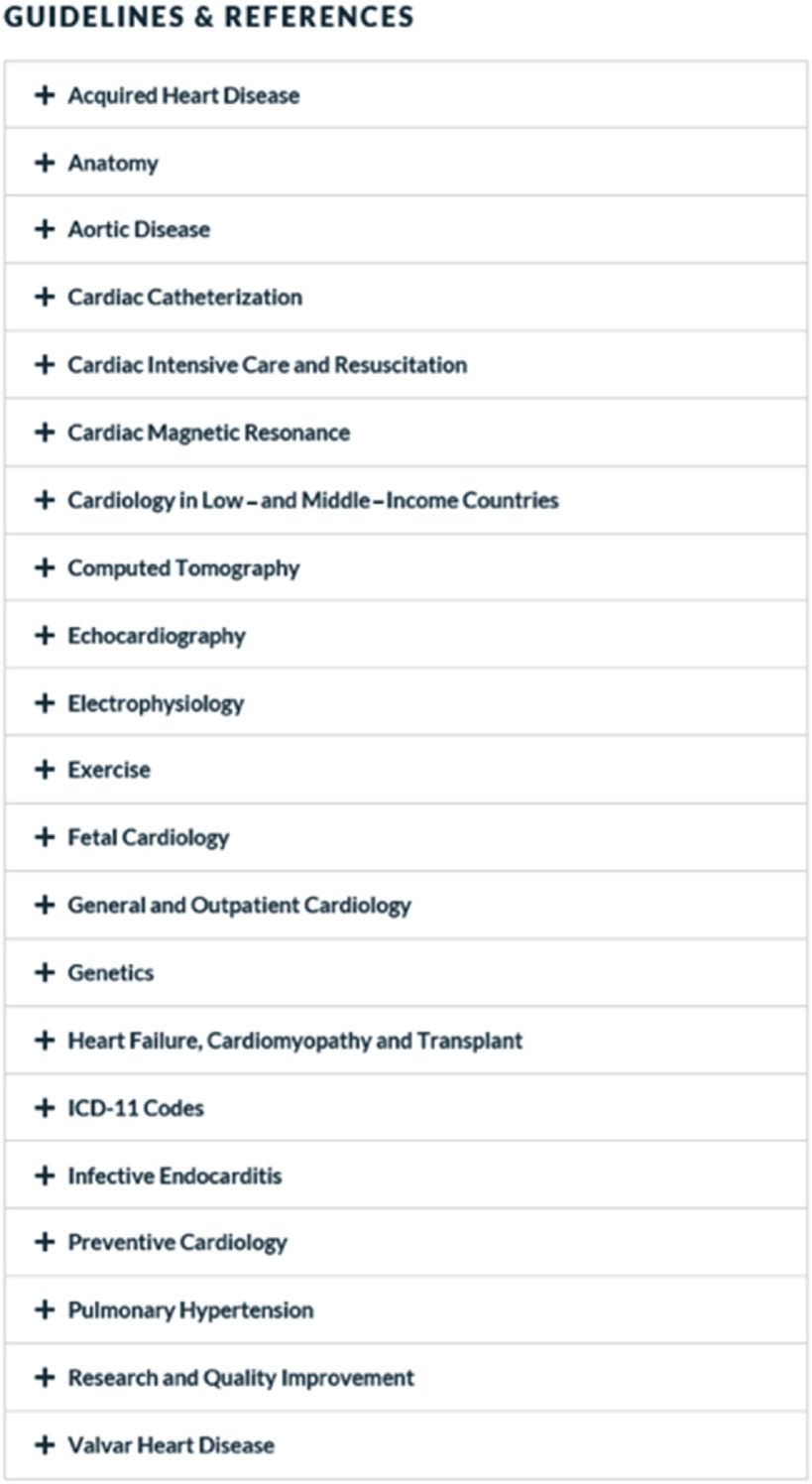
Guidelines and References listing on the Pediatric Cardiac Learning Center.

In addition to this curated collection of learning modules and conference recordings, a developing library of practice guidelines and key references is easily accessible on both the Pediatric Cardiac Learning Center (Fig 8) and ACHD Learning Center, and a calendar of applicable upcoming conferences and events is displayed on both component websites.

### Editorial board, content development team leaders and content developers

Simultaneous with the development of the website was the organisation of existing content from the ACHD Learning Center, as well as creation and acquisition of new content for both component sites. To fulfil the aims of keeping a high standard of accurate and quality content, making certain it meets the needs of the modern-day adult learner, covering the breadth of required knowledge for providers of congenital and paediatric-acquired heart disease, and catering to a global audience, it was paramount to involve providers with a deep and broad range of expertise from around the world.

The editorial leadership of the Pediatric Cardiac Learning Center followed the example of the already existing ACHD Learning Center Editorial Board, by seeking and selecting a well-rounded international group of experts covering a wide range of subspecialties. Additionally, it was crucial to provide not only experts in the applicable medical content, but also experts in medical education of adult learners, including fellowship programme directors actively involved in trainee fellowship programmes in various international settings. The Editorial Board is responsible for quality review, curation, and organisation of content. Content teams were also organised, with content team leaders and team members from around the world. The content team leaders are responsible for creating a desired curriculum, identifying gaps in current content, working with content team members to create and review needed content, as well as identifying existing evidence-based content that fulfilled the quality expectation and needs of the outlined curriculum. Fulfilling the aim for global representation, there are currently 27 countries represented across the Pediatric Cardiac Learning Center and ACHD Learning Center Editorial Boards and content team leaders.

### Organisational endorsement

From its inception, the International Society for Adult Congenital Heart Disease was the official endorsing organisation of the ACHD Learning Center. The involvement of the largest international organisation in the field of ACHD was clearly a key to the early global success of the ACHD Learning Center. Following suite, the Pediatric Cardiac Learning Center has partnered with the Association for European Paediatric and Congenital Cardiology as the official endorsing organisation for this component site. The two organisations not only are intimately involved in their respective sites with International Society for Adult Congenital Heart Disease and Association for European Paediatric and Congenital Cardiology members present on the Editorial Boards and content creation teams, but also in providing conference recordings. Children’s HeartLink is endorsing and will assist with content development for the cardiology in low- and middle-income countries lecture series. This material will be provided to all users of Heart University, but will specifically be used for supplemental education for the Fellowship in Pediatric Cardiac Sciences in Low- and Middle-Income Countries supported by Children’s HeartLink. There are ongoing discussions regarding official endorsement from other major specialty organisations worldwide, with the aim that Heart University will be validated as the major online educational forum in our field.

### Partnering with the International Society for Nomenclature of Paediatric and Congenital Heart Disease: the “IPCCC ICD-11 Congenital Heart Atlas”

In December 2019, Heart University officially partnered with the International Society for Nomenclature of Paediatric and Congenital Heart Disease (the Nomenclature Society) to provide a new online framework for the portrayal and further development of the Nomenclature Society’s Congenital Heart Atlas linked to the 11th revision of the International Classification of Diseases (ICD-11) and the International Paediatric and Congenital Cardiac Code (IPCCC).

The Nomenclature Society was conceived in the early 2000s to standardise nomenclature for paediatric and CHD.^14^ Since then, the society has developed and maintained the IPCCC.^15^ In the last decade, the Nomenclature Society launched projects to define and illustrate the IPCCC^16,17^ by constructing an online Atlas of Congenital Heart Disease, initially limited to anatomical photographs of cardiac specimens. The initial version of this web-based “Global Virtual Museum of Congenital Cardiac Pathology” was funded via a research grant from the Children’s Heart Foundation.^16,17^ Recently, the Nomenclature Society published a consensus document of nomenclature for congenital cardiac disease, which was adopted by the World Health Organization as the paediatric and congenital cardiac component of ICD-11.^18^

In partnership with Heart University, the Atlas project of the Nomenclature Society will now be expanded to illustrate the CHD terms of ICD-11 on an enhanced platform that will include multiple imaging modalities. The “*IPCCC ICD-11 Congenital Heart Atlas*” will become a third component site of Heart University, providing an easily navigable list of hierarchical terms from the IPCCC, with definitions, synonyms, and representative images. The images will be based on the IPCCC ICD-11 coding system and will include photographs and videos of anatomic specimens, intraoperative photography, anatomic diagrams and cartoons, and photographs and videos from any relevant imaging modality. The goal will be for this consensus terminology to be incorporated into the Pediatric Cardiac Learning Center and ACHD Learning Center, with seamless linking between the component sites.

### Partnering with the World Congress of Pediatric Cardiology and Cardiac Surgery

The Eighth Quadrennial World Congress of Pediatric Cardiology and Cardiac Surgery will be held in Washington, District of Columbia, United States, from 19 September, 2021 to 24 September, 2021 [http://www.wcpccs2021.org/]. The Quadrennial World Congress of Pediatric Cardiology and Cardiac Surgery truly represents “The Olympics of Our Profession”.^19,20^ Heart University and The World Congress will partner to record Landmark Lectures and other key academic content at the Eighth World Congress. As a global service to the community of paediatric and congenital cardiac care, this content will be available free of charge via the internet at Heart University to anyone interested.

### Partnering with the Cardiology in the Young

*Cardiology in the Young* [https://www.cambridge.org/core/journals/cardiology-in-the-young] is devoted to all aspects of paediatric and congenital cardiac care, including cardiovascular issues affecting the young as well as older patients with CHD or illness secondary to other cardiac diseases acquired during childhood. The journal serves the interests of all professionals concerned with paediatric and congenital cardiac care. By design, the journal is international and multidisciplinary in its approach, and members of the Editorial Board take an active role in its mission, helping to make it an indispensable reference for paediatric and congenital cardiac care.

*Cardiology in the Young* is the Official Journal of the following three organisations: 1) Association for European Paediatric and Congenital Cardiology [https://www.aepc.org/about-aepc], 2) The International Society for Nomenclature of Paediatric and Congenital Heart Disease [http://ipccc.net/], and 3) The Quadrennial World Congress of Pediatric Cardiology and Cardiac Surgery [http://www.wcpccs2021.org/]^21^ Heart University already has important partnerships with all three of these organisations as well; and therefore, it is natural that Heart University and *Cardiology in the Young* will partner. This White Paper that you are now reading represents one component of the multiple components of the partnership between Heart University and *Cardiology in the Young*. Several members of the Editorial Boards of Heart University have been appointed to the Editorial Board of *Cardiology in the Young*, including Justin T. Tretter,Rohit S. Loomba, and Andrew N. Redington. These common members of the Editorial Boards will assure ongoing alignment of the missions of Heart University and *Cardiology in the Young*. Another area of collaboration is that Heart University will begin to feature White Papers, Guideline Papers, and Landmark Papers published in Cardiology in the Young.^18,22,23^ Approximately every 4 months, *Cardiology in the Young* will provide Heart University with a new “hot of the press” major paper that is published in *Cardiology in the Young*. Heart University will post and highlight this paper on the Heart University site. A discussion board surrounding the paper will be established and the lead author of the manuscript will be invited to moderate the discussion. Finally, bidirectional hyperlinks will be established between the websites of Heart University and *Cardiology in the Young*. The leadership of Heart University and *Cardiology in the Young* are currently actively exploring additional opportunities for collaboration.

### Other key partnerships

One aim for Heart University is to create a centralised, open access source of applicable information. While we aim to create comprehensive trainee curriculums and collections of recorded conference topic material, we do not aim to “recreate the wheel”. In this light we have made key partnerships with other topical sites, networks, and podcasts with hyperlinked logos displayed in our Additional Resources section, along with a separate section highlighting features of the Congenital Heart International Professionals Network (CHiP Network), for example.

## Current standing and future goals

A “soft launch” for the newly designed Heart University was undertaken in February 2019, soliciting the site during the 22^nd^ Annual Update on Pediatric and Congenital Cardiovascular Disease. The site was further promoted during the annual Association for European Paediatric and Congenital Cardiology and International Society for Adult Congenital Heart Disease conferences during this same year. From February 2019 to June 2019, user feedback was collected and used to improve upon the organisation, graphical design, and navigation of the site. During this trial period, the number of registered users increased to 3200 users from 75 countries and 6 continents. The redesign of the site was completed January 2020, followed by an official “hard launch”.

We continue to collect, create, and curate training modules with test questions to complete comprehensive curriculums for trainees in paediatric cardiology and ACHD. Other learning curriculums are in development, including curriculums specific for nurses. A clear limitation to providing a large amount of material readily available online with no structured plan for navigating through the material and assessing mastery of the content is that the user may become complacent, with little motivation to work comprehensively through the outlined curriculums. The end result is a broad superficial familiarity with a topic without a mastery of its fundamental concepts. We are working to build in some rigour into the process of online learning through certification courses that mandate answering questions relating to the topic material (i.e., Certification in Cardiac Anatomy). The site eventually aims to provide continued medical education credit for practising providers, navigating the various specific international requirements. We additionally are actively pursuing a means to provide objective credit and applicable analytical feedback for content creators of learning modules that will be pertinent for career advancement. While our focus is to continue to improve upon and fill the gaps of the current three component sites, we have also been in discussion with other international organisations wishing to create component sites specific to their specialties, e.g., congenital cardiac surgery and cardiac anaesthesia.

While we have accomplished much from the inception of Heart University in 2017 to the present, there remains much to be done, and as with any form of education, it will never be complete. However, we are convinced that today and tomorrow’s learner (which is all of us) will benefit from this unique and comprehensive resource. The textbook is not yet dead, and we hope it never will be, but online learning and access to educational material is already a key component of our everyday lives, and one that will only increase in the future. With the current coronavirus pandemic, there has never been more urgency than now to establish options for supplemental online learning. We owe a huge debt of gratitude to all those that have given their time, expertise, and support to make Heart University what it is today and welcome anyone who wishes to join us and contribute to the future of this exciting project.
